# Supplementary material for: Evidence supported by Mendelian randomization: impact on inflammatory factors in knee osteoarthritis
Source: Front Med (Lausanne). 2024 May 28;11:1382836. doi: 10.3389/fmed.2024.1382836 (PMC11165061; doi:10.3389/fmed.2024.1382836)
Supplement: Supplementary file 1 [file Data_Sheet_1.ZIP › data/GCST90274833_5e6/833.forest.pdf]

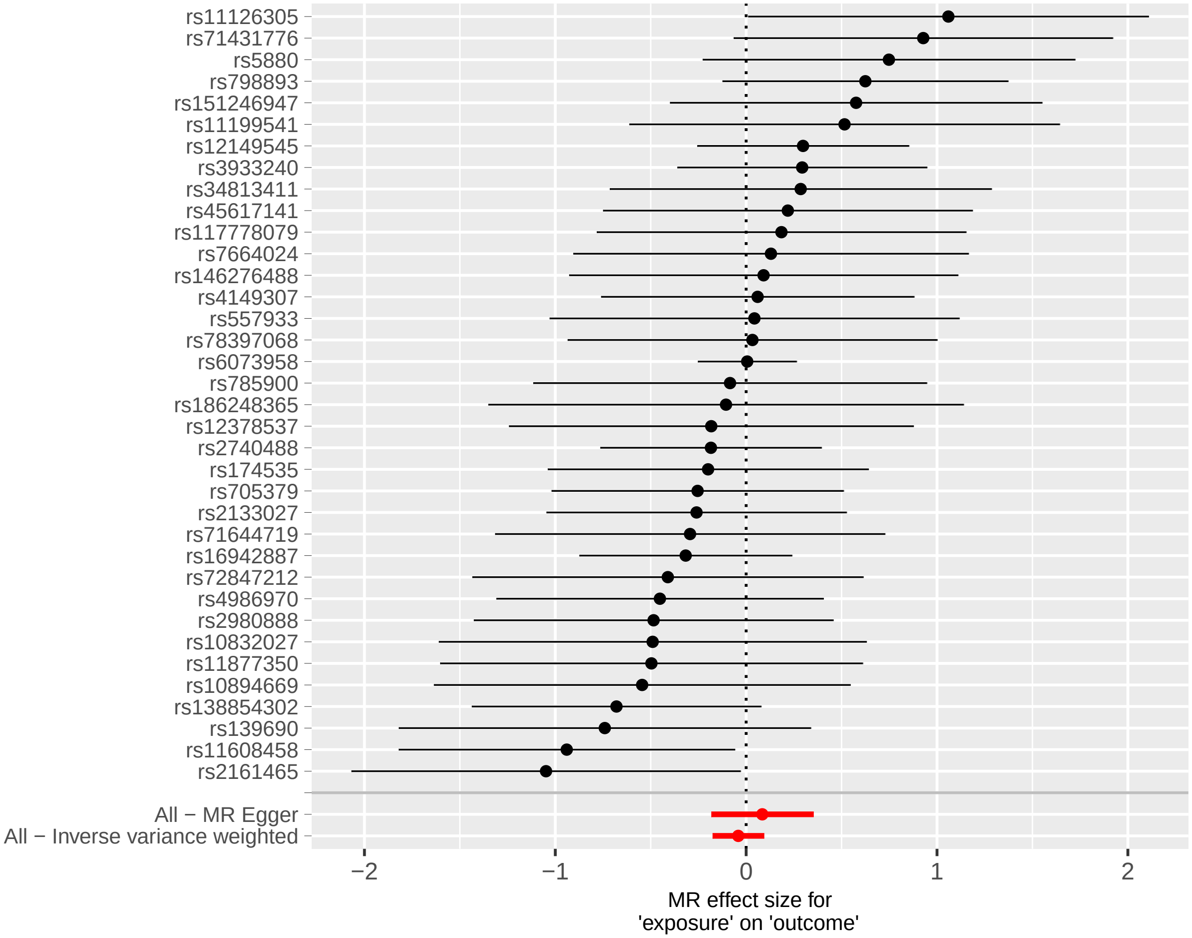
rs11126305
rs71431776
rs5880
rs798893
rs151246947
rs11199541
rs12149545
rs3933240
rs34813411
rs45617141
rs117778079
rs7664024
rs146276488
rs4149307
rs557933
rs78397068
rs6073958
rs785900
rs186248365
rs12378537
rs2740488
rs174535
rs705379
rs2133027
rs71644719
rs16942887
rs72847212
rs4986970
rs2980888
rs10832027
rs11877350
rs10894669
rs138854302
rs139690
rs11608458
rs2161465
All − MR Egger
All − Inverse variance weighted
−2
−1
0
1
2
MR effect size for
'exposure' on 'outcome'
